# Assessment of symptom improvement following nasal septoplasty with or without turbinectomy

**DOI:** 10.1590/S1808-86942011000500007

**Published:** 2015-10-22

**Authors:** Leandro Castro Velasco, Lisandra Megumi Arima, Romualdo Suzano Louzeiro Tiago

**Affiliations:** 1 Medical resident (R3) at the Otorhinolaryngology Unit, HSPM; 2 Medical resident (R3) at the Otorhinolaryngology Unit, HSPM; 3 Doctoral degree in sciences, São Paulo Federal University. Post-doctoral degree, São Paulo Federal University. Assisting physician of the Otorhinolaryngology Unit, HSPM

**Keywords:** nasal septum, signs and symptoms, therapeutics, turbinates

## Abstract

**Abstract:**

Most studies show that objective measures to quantify and determine surgical success in the treatment of nasal obstruction do not correlate with subjective improvement as reported by patients.

**Aim:**

To evaluate the subjective improvement of nasal symptoms in patients undergoing septoplasty with or without turbinectomy.

**Materials and methods:**

A prospective study. We evaluated 72 septoplasty patients with or without partial inferior turbinectomy; the patients answered a questionnaire preoperatively and on the 60th day after surgery.

**Results:**

Septoplasty was done associated with bilateral partial inferior turbinectomy in 83.3% of patients; it was unilateral in 9.7%; there was no need for turbinate reduction in 6.9%. An improvement of all symptoms was observed after surgery. Nasal obstruction had improved in 68 patients (94.4%) by the 60th postoperative day. The average nasal obstruction score in patients with and without allergic symptoms was similar before surgery and on the 60th postoperative day. Older patients had milder preoperative allergic symptoms.

**Conclusions:**

Nasal symptoms in patients undergoing septoplasty, with or without turbinectomy, improved. Patients with and without allergic symptoms showed a similar improvement of nasal obstruction on the 60th postoperative day.

## INTRODUCTION

Nasal obstruction is one of the main symptoms in an ENT specialist's practice; nasal septum deviation is one of its most frequent causes. Other causative conditions are: adenoid hypertrophy; turbinate hypertrophy; nasal tumors; and nasal polyps^1^. At present in the United States, 5 billion dollars are spent on medication to provide relief from nasal obstruction^2^.

Nasal septum corrective surgery (septoplasty) was started in the 19^th^ century, and has been modified and improved since. The techniques have attempted to provide maximum functional and respiratory improvement at the same time preserving other physiologic functions of the nose (filtering, warming, and moisturizing the air) to improve nasal flow^3^. In the US, a septoplasty is the third most common procedure in otorhinolaryngology; it aims to improve the quality of life^4^.

Hypertrophy of the lower turbinates may also cause nasal obstruction by occluding the nasal valve^1^. There are several surgical techniques for treating turbinate hypertrophy, such as partial or total turbinectomy, turbinoplasty, submucosal or extramucosal electrocautery, and resection by radiofrequency, laser, or cryotherapy^5^. These techniques aim to reduce the volume of the lower turbinate to improve nasal obstruction and preserve nasal function. There is currently no consensus in the literature about which turbinate-reducing technique is best^5^.

Objective measures to quantify and establish the success of surgery for nasal obstruction have been a challenge. Several methods have been proposed; the two most common are rhinomanometry and acoustic rhinometry. Rhinomanometry measures nasal flow resistance during breathing. Acoustic rhinometry measures nasal permeability and quantifies the cross-sectional area of the nostrils up to the nasopharynx, as well as the nasal cavity volume between any two chosen cross-sections^6^. Most studies have shown that these methods do not correlate with the patient's reported subjective improvement^6^,^7^. A few studies have shown, however, that septoplasty is generally effective for treating nasal obstruction, and that most patient show improvements in nasal symptoms^1^,^2^,^4^,^7^, ^8^, ^9^, ^10^, ^11^.

## OBJECTIVE

The purpose of this study was to assess the improvement of nasal symptoms in patients undergoing septoplasty with or without turbinectomy.

## MATERIAL AND METHODS

The sample comprised 72 patients that were seen at the otorhinolaryngology unit of a tertiary hospital in Sao Paulo, SP. These patients had been diagnosed with nasal septum deviation with or without lower turbinate hypertrophy. Patients were treated medically with antihistamines and topical corticosteroids. Following this approach, patients that did not improve underwent septoplasty with or without turbinectomy. Surgeries were done consecutively from May 2009 to June 2010. The institutional review board approved the study (Report no. 19/2010).

Patients with nasal septum deviation with unilateral or bilateral chronic nasal obstruction and symptoms persisting after two months of medical therapy (topical corticosteroids with or without antihistamines) were enrolled for this study.

The following conditions were exclusion criteria: septoplasty with nasosinusal surgery; nasosinusal tumors; chronic rhinosinusitis; rhinoplasty; head and neck radiotherapy; perforated nasal septum; insufficient nasal valve; nasosinusal granulomatous disease; hyperplasic pharyngeal tonsils; snoring surgery; craniofacial malformation; and pregnancy.

A longitudinal prospective cohort study was undertaken in which patients were assessed preoperatively and on the 7^th^, 14^th^, 30^th^, and 60^th^ postoperative day. This evaluation consisted of an otorhinolaryngological examination, a questionnaire with questions on the main symptoms of nasal obstruction (nasal obstruction, coryza, pruritus, sneezing, facial pain, snoring, sleep disorders, daytime drowsiness), and a score of each. The intensity of symptoms was scored from 1 to 4, as follows: 1 – absence of symptoms; 2 – mild symptoms; 3 – moderate symptoms; 4 – severe symptoms. Data were gathered on sex, age, and degree of septal deviation. Cottle's classification^12^ was used to classify the site and degree of septal deviation. Lower turbinate hypertrophy was graded as: normotrophic; mild hypertrophy (grade 1); moderate hypertrophy (grade 2), and severe hypertrophy (grade 3). Nasal turbinates were reduced in patients with hypertrophy grade 2 (43 patients) and 3 (24 patients). Second year medical residents – supervised by assisting physicians from our unit – performed the procedures. General anesthesia was done using the modified Cottle's technique^3^. Partial inferior turbinectomy with direct photophore visualization was the procedure done on the lower turbinate. Bilateral partial inferior turbinectomy was done in 60 patients; unilateral partial inferior turbinectomy was done in seven patients; no turbinectomy was done in five patients. Among the turbinectomy patients, 23 required postoperative nasal packing for 24 to 72 hours; electrocautery hemostasis on the operative site was sufficient in 44 patients. Nasal splints were used in 69 patients from 7 to 10 days. Al patients were asked to perform nasal cavity irrigation with saline and cephalexin on the first seven postoperative days.

The sample was allocated to two groups: patients with nasal obstruction and allergy symptoms, and patients with nasal obstruction without allergy symptoms. Patients without allergy symptoms were those that had no pruritus, sneezing, or coryza – grade 1 for these three symptoms. Patients with allergy symptoms had these three symptoms graded as 2 or more, or two of the three symptoms (coryza, sneezing, and pruritus) simultaneously graded 2 or more. Patients that did not fit into these two categories were not included in the comparison of groups with or without allergy symptoms.

The following statistical methods were used in the analysis: analysis variance (ANOVA), and the independent t test. Statistical significance was *p* ≤ 0.05.

## RESULTS

The study sample comprised 72 patients Chart 1), of which 30 (41.7%) were female and 42 (58.3%) were male. The mean age was 34.49 years, ranging from 9 to 68 years.

**Chart 1:** Mean nasal symptom scores (nasal obstruction, facial pain, nasal pruritus, sneezing bouts/sneezing, coryza, snoring, daytime drowsiness, and sleep disorders) in the preoperative (Pre) and postoperative (PO) periods.

SYMPTOMS		Pre-op	7^th^ PO	14^th^ PO	30^th^ PO	60^th^ PO	ANOVA (*p*)	RESULTS
Nasal obstruction	Mean ±DP	3.5 ±0.6	1.7 ±0.8	1.6 ±0.8	1.4 ±0.6	1.3 ±0.6	<0.001	Pre > 7^th^ PO = 14^th^ PO > 30^th^ PO = 60^th^ PO
Facial pain	Mean ±DP	1.7 ±1.0	1.5 ±0.7	1.4 ±0.6	1.2 ±0.5	1.2 ±0.6	<0.001	Pre > 7^th^ PO = 14^th^ PO > 30^th^ PO = 60^th^ PO
Nasal pruritus	Mean ±DP	2.4 ±1.2	1.4 ±0.6	1.6 ±0.8	1.6 ±0.7	1.4 ±0.6	<0.001	Pre > 7^th^ PO = 14^th^ PO > 30^th^ PO = 60^th^ PO
Sneezing	Mean ±DP	2.4 ±1.2	1.5 ±0.7	1.6 ±0.7	1.6 ±0.7	1.5 ±0.7	<0.001	Pre > 7^th^ PO = 14^th^ PO > 30^th^ PO = 60^th^ PO
Coryza	Mean ±DP	2.1 ±1.2	1.8 ±0.8	1.6 ±0.7	1.5 ±0.7	1.4 ±0.6	<0.001	Pre > 7^th^ PO = 14^th^ PO > 30^th^ PO = 60^th^ PO
Snoring	Mean ±DP	2.6 ±1.2	1.9 ±1.0	1.5 ±0.7	1.6 ±0.8	1.5 ±0.7	<0.001	Pre > 7^th^ PO = 14^th^ PO > 30^th^ PO = 60^th^ PO
Drowsiness	Mean ±DP	2.0 ±1.1	1.4 ±0.8	1.1 ±0.3	1.1 ±0.3	1.0 ±0.2	<0.001	Pre > 7^th^ PO = 14^th^ PO > 30^th^ PO = 60^th^ PO
Disordered sleep	Mean ±DP	1.8 ±1.1	1.4 ±0.8	1.2 ±0.5	1.1 ±0.4	1.1 ±0.3	<0.001	Pre > 7^th^ PO = 14^th^ PO > 30^th^ PO = 60^th^ PO

SD = standard deviation; number of patients = 72; *p* ≤0.05 (ANOVA)

In our sample, bilateral partial inferior turbinectomy was done in 83.3% of patients, unilateral partial inferior turbinectomy was done in 9.7% of patients, and no turbinectomy was done in 6.9% of patients. As the number of patients that did not require partial inferior turbinectomy was small (five patients), this group was not compared with groups of patients undergoing turbinectomy.

Symptoms regressed postoperatively in all patients; the preoperative and postoperative difference was statistically significant (Chart 1). Analysis of nasal obstruction and facial pain showed that there was a marked improvement between the preoperative period and the 7^th^ postoperative day (*p*<0.001), no improvement between the 7^th^ and 14^th^ postoperative days, followed by a second improvement between the 14^th^ and 30^th^ postoperative days (*p*<0.001), reaching a plateau thereafter until the 60^th^ postoperative day (Chart 1).

There was significant improvement of nasal pruritus and bouts of sneezing between the preoperative period and the 7^th^ postoperative day (*p*<0.001), and no further improvement between the 7^th^ and 60^th^ postoperative days (Chart 1). There was marked improvement in coryza between the preoperative period and the 7^th^ postoperative day (*p*<0.001), followed by improvement between the 7^th^ and 14^th^ postoperative days (*p*<0.001), no additional improvement between the 14^th^ and 30^th^ postoperative days, and additional improvement between the 30^th^ and 60^th^ postoperative days (Chart 1).

Snoring, sleep disorders, and daytime drowsiness regressed significantly in patients between the preoperative period and the 7^th^ postoperative day (*p*<0.001), followed by further improvement between the 7^th^ and 14^th^ postoperative days (*p*<0.001), and no added improvement between the 14^th^ and the 60^th^ postoperative days (Chart 1).

Nasal obstruction regressed in 68 patients (94.44%) on the 60^th^ postoperative day compared to the preoperative period (green); it remained unchanged in one patient (1.4%) (yellow); and it worsened in three patients (4.2%) (red), as shown on Table 1.

**Table 1:** Absolute and relative frequency of patients with nasal obstruction in the preoperative (Pre) and postoperative (60° PO) periods according to the degree of obstruction.

			PRE				Total
			1	2	3	4	
		n	0	1	23	35	59
	1						
		%	-	1.4%	31.9%	48.6%	81.9%
	2	n	0	0	6	2	8
		%	-	-	8.3%	2.8%	11.1%
60^th^ PO							
	3	n	0	2	1	1	4
		%	-	2.8%	1.4%	1.4%	5.6%
	4	n	0	0	1	0	1
		%	-	-	1.4%	-	1.4%
Total		n	0	3	31	38	72
		%	-	4.2%	43.0%	52.8%	100%

n = frequency of patients.

**Figure 1:**
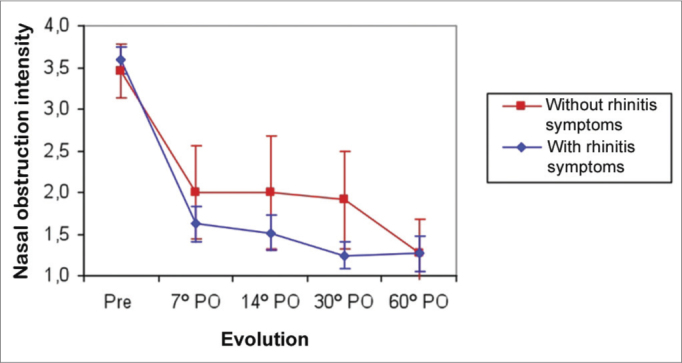
Mean nasal obstruction score in patients with or without allergic symptoms in the preoperative (Pre) and postoperative (PO) periods.

Nasal obstruction in patients with and without allergy symptoms regressed significantly between the pre-operative period and the 7^th^ postoperative day (*p*<0.001), followed by a stable condition between the 7^th^ and 14^th^ postoperative days in both groups. Patients with allergy symptoms improved between the 14^th^ and 30^th^ postoperative days (*p*<0.001), and then stabilized between the 30^th^ and 60^th^ postoperative days. Patients with no allergy symptoms remained stable between the 14^th^ and 30^th^ postoperative days, and them improved further between the 30^th^ and 60^th^ postoperative days (*p*<0.001). The mean nasal obstruction score in both groups was similar in the preoperative period and on the 7^th^ postoperative day. The mean score was higher in the group without allergy symptoms between the 14^th^ and 30^th^ postoperative days (*p*<0.02). The mean scores were similar in both groups on the 60^th^ postoperative day (Figure 1).

The sample was divided into three age groups: <20 years (Group 1); 20 to <40 years (Group 2), and ≥40 years (Group 3). Patients in the three groups developed similarly in terms of nasal obstruction, facial pain, disordered sleep, and daytime drowsiness. There were no differences in the mean scores of preoperative and postoperative nasal obstruction and facial pain among the three groups.

**Figure 2:**
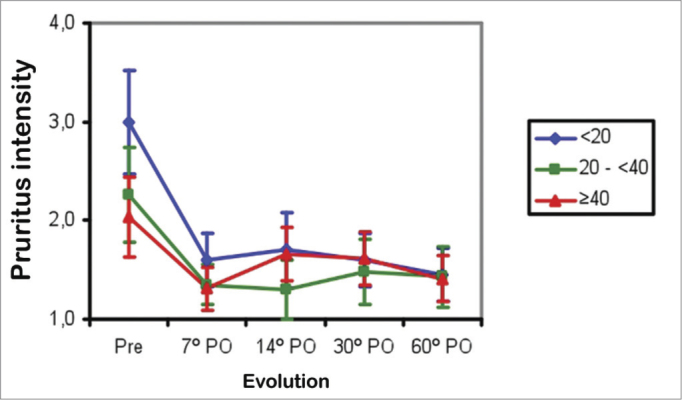
Mean pruritus score in three age groups (years) in the preoperative (Pre) and postoperative (PO) periods.

In Groups 1 and 2 patients, pruritus improved between the preoperative period and the 7^th^ postoperative day (*p*<0.001), and thereafter remained unchanged (from the 7^th^ to the 60^th^ postoperative days. In Group 3, pruritus improved between the preoperative period and the 7^th^ postoperative day, then worsened between the 7^th^ and 14^th^ postoperative days (*p*<0.001), and remained unchanged between the 14^th^ and the 60^th^ postoperative days. The mean pruritus score was higher in Group 1 compared to Group 2 (*p*<0.02), and similar between Groups 2 and 3 in the preoperative period. The mean score was similar in the three groups between the 7^th^ and 60^th^ postoperative days (Figure 2).

**Figure 3:**
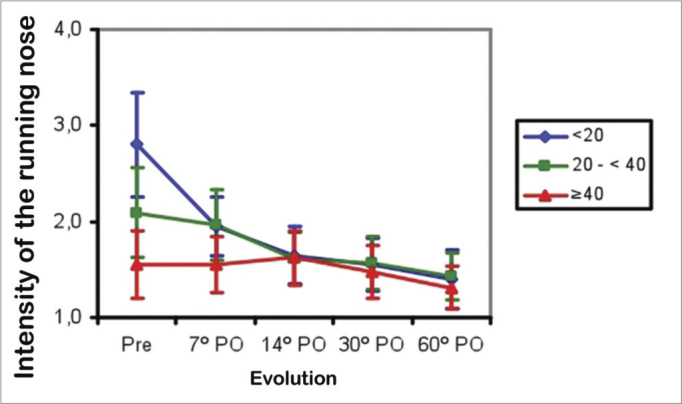
Mean coryza score in three age groups (years) in the preoperative (Pre) and postoperative (PO) periods.

Coryza improved in Group 1 patients between the preoperative period and the 7^th^ postoperative day (*p*<0.001), then remained unchanged between the 7^th^ and 60^th^ postoperative days. In Group 2, coryza did not improve between the preoperative period and the 7^th^ postoperative day, then improved between the 7^th^ and the 14^th^ postoperative days (*p*<0.001), and remained unchanged between the 14^th^ and the 60^th^ postoperative days. In Group 3, coryza did not improve between the preoperative period and the 60^th^ postoperative day. The mean score for coryza among groups was higher in Group 1 compared to Group 2, and higher in Group 2 compared to Group 3 in the preoperative period *(p*<0.001). The mean scores were similar between the 7^th^ and 60^th^ postoperative days in the three groups (Figure 3).

**Figure 4:**
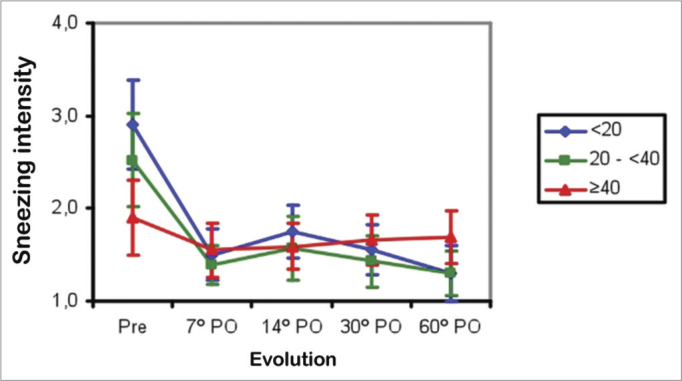
Mean bouts of sneezing/sneezing score in three age groups (years) in the preoperative (Pre) and postoperative (PO) periods.

Sneezing bouts in Groups 1 and 2 patients improved between the preoperative period and the 7^th^ postoperative day (*p*<0.001), then remained unchanged between the 7^th^ and 60^th^ postoperative days. In Group 3, patients showed no improvements in the mean scores of sneezing bouts between the preoperative period and the 60^th^ postoperative day. The mean sneezing bout scores were similar in Groups 1 and 2, and were both higher compared to Group 3 in the preoperative period (*p*<0.001). These means became similar in all groups between the 7^th^ and the 60^th^ postoperative days (Figure 4).

**Figure 5:**
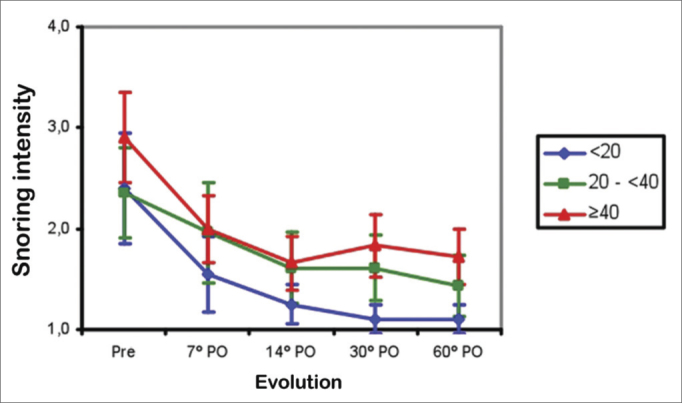
Mean snoring score in three age groups (years) in the preoperative (Pre) and postoperative (PO) periods.

Snoring improved significantly in Groups 1 and 2 patients between the preoperative period and the 7^th^ postoperative day (*p*<0.001), then improved further between the 7^th^ and 14^th^ postoperative days (*p*<0.001), and thereafter remained unaltered between the 14^th^ and 60^th^ postoperative days. Snoring improved between the preoperative period and the 7^th^ postoperative day in Group 3 (*p*<0.001). The mean snoring score was similar in all groups between the preoperative period and the 14^th^ postoperative day. Between the 30^th^ and 60^th^ postoperative days, the mean snoring score was lower in Group 1 compared to Group 2 (*p*<0.001), and similar between Groups 2 and 3 (Figure 5).

The symptoms nasal obstruction, coryza, nasal pruritus, snoring, disordered sleep, and daytime drowsiness progressed similarly in male and female patients. There was no difference in the mean nasal obstruction score between both sexes in the preoperative and postoperative periods.

**Figure 6:**
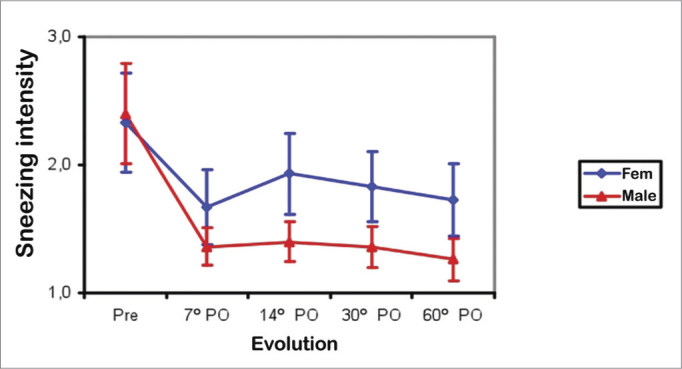
Mean coughing bout score in males (Male) and females (Fem) in the preoperative (Pre) and postoperative (PO) periods.

Sneezing bouts improved similarly in male and female patients; there was significant improvement between the preoperative period and the 7^th^ postoperative day (*p*<0.001), then no further improvement between the 7^th^ and 60^th^ postoperative days. The mean sneezing bout scores were similar in both sexes in the preoperative period. The mean score was higher in females between the 7^th^ and 60^th^ postoperative days (Figure 6).

**Figure 7:**
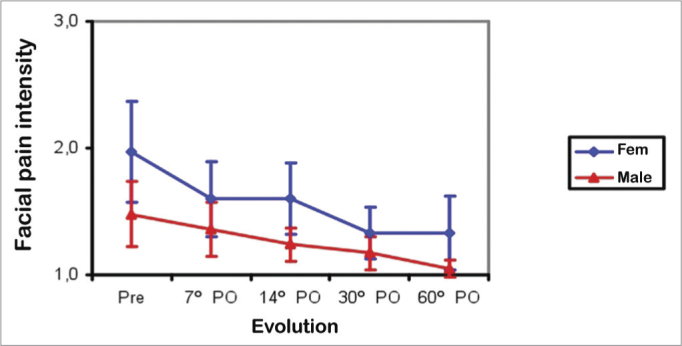
Mean facial pain score in males (Male) and females (Fem) in the preoperative (Pre) and postoperative (PO) periods.

Facial pain improved similarly in male and female patients between the preoperative period and the 7^th^ postoperative day (*p*<0.002); there was no further improvement between the 7^th^ and 60^th^ postoperative days. The mean facial pain score was higher in females than males in the preoperative period, and between the 14^th^ and 60^th^ postoperative days (*p*<0.04). The mean intensity scores were similar in both groups between the 7^th^ and 30^th^ postoperative days (Figure 7).

A comparison of nasal septum deviation (grades 2 or 3) with mean nasal obstruction scores in the preoperative period and the 60^th^ postoperative day showed that there was a significant improvement between the preoperative period and the 60^th^ postoperative day in both groups (*p*<0.0001). On the other hand, there was no difference in the nasal septum deviation grade between the preoperative period and the 60^th^ postoperative day.

## DISCUSSION

Septoplasty is one of the most frequently done surgical procedures worldwide. Several studies have shown the benefit of this procedure; most of them, however, are retrospective studies^2^. These studies consisted of registry reviews or questionnaires by telephone^1^. Other retrospective studies have applied quality of life questionnaires not focusing specifically on nasal obstruction^2^. The present study was prospective and focused on an evaluation of the main nasal symptoms, including nasal obstruction. We found that septoplasty, with or without turbinectomy, resulted in improvement of all symptoms that we studied (Chart 1). Symptoms regressed more markedly within the first seven postoperative days; then patients improved more gradually until the 60th postoperative day with regards to most symptoms. However, we found that the symptoms sneezing and nasal pruritus regressed significantly on the first evaluations (7^th^ day) and thereafter stabilized until the 60^th^ postoperative day. We believe that removing the nasal splint and avoiding contact between the mucosa of the nasal septum and the turbinates were the main improvement factors in the first evaluation.

Surgical success may be established by objective and/or subjective measures. The main objective measures were made with rhinomanometry and acoustic rhinometry^6^,^7^,^10^. A few studies have shown that these tests are unrelated with the patient's subjective improvement^6^,^7^,^10^,^13^. Therefore, we chose not to use objective methods to measure changes in symptoms.

Prospective studies have reported high satisfaction scores after septoplasty. The reported success rate in the literature range from 63% to 89.5% depending on the duration of follow-up and the method for evaluating results^4^,^11^. The subjective improvement rate of nasal obstruction in our sample was 94.4% (Table 1); there were no age differences in this improvement. Gandomi et al.^4^ have stated that the improvement rate might be related with age. Younger patients tend to benefit more from surgery due to anatomic rather than dynamic factors. The mean age in our sample was 34.5 years, which is higher than the mean age in Gandomi et al.'s study^4^, in which the mean age was 22.4 years; it is also lower than the majority of other papers, in which the mean age was above 40 years^1^. One of the reasons for the higher satisfaction rate in our study may be a 60-day follow-up, which is shorter compared to other studies^1^,^4^,^13^.

Stewart et al.^1^ and Gandomi et al.^4^ have shown that nasal obstruction improves significantly within the first three postoperative months, and that this symptom stabilizes by six months after surgery. Jessen et al.^14^ concluded that an initial improvement of nasal obstruction in the first months to years after surgery becomes progressively undervalued, especially when other causes of obstruction are present, such as chronic rhinitis and sinusitis^1^,^4^. In our sample, the improvement was significant between the preoperative period and the 7^th^ postoperative day, followed by a mild improvement between the 14^th^ and 30^th^ postoperative day, which remained constant 60 days after surgery (Chart 1).

About 93% of our patients underwent partial inferior turbinectomy – it was bilateral in 83.3% and unilateral 9.7% of cases. Partial inferior turbinectomy is used to relieve nasal obstruction in patients with hypertrophic inferior turbinates or allergic or non-allergic causes^15^. Bilateral partial inferior turbinectomy, with or without septoplasty, relieves coryza, pruritus, sneezing, and nasal obstruction^15^. Bandos et al.^16^ have shown that 90% of patients undergoing partial inferior turbinectomy had partial or full improvement of nasal obstruction between surgery and the first 12 months postoperatively. However, only 30% of patients reported partial or total relief of nasal obstruction two years after surgery. In our sample, all allergy symptoms improved significantly between the preoperative period and the 7^th^ postoperative day (Chart 1), and progressed differently after this period. These symptoms may have decreased because inferior turbinectomy reduced the exposed mucosal surface and gland tissue to allergens^15^.

Nasal obstruction is common in patients with sleep apnea syndrome – which causes fragmented sleep, daytime drowsiness, and loss of quality of life. Septoplasty may reduce snoring and daytime drowsiness in patients with the sleep apnea/hypopnea syndrome^4^,^17^. Snoring, disordered sleep, and daytime drowsiness regressed significantly after surgery, and remained improved on the 60^th^ postoperative day (Chart 1) in our sample.

Patients with allergy symptoms that underwent septoplasty improved more of nasal obstruction by the 30^th^ postoperative day compared to patients without allergy symptoms; the improvement because similar by the 60^th^ postoperative day (Figure 1). This is probably due to more significant inferior turbinate hypertrophy in patients with allergy symptoms, which requires wider resection of the turbinates and therefore relieves nasal obstruction more abruptly.

Nasal obstruction, pruritus, facial pain, snoring, sleep disorders, and daytime drowsiness improved in all age ranges in the three groups. Older patients had less intense allergy symptoms (coryza, sneezing, and pruritus) preoperatively (Figures 2 to 4). Coryza and sneezing bouts did not improve postoperatively in patients aged over 40 years (Figures 3 and 4). This is probably because the incidence of allergic rhinitis is higher in younger patients^18^,^19^.

Several factors give rise to snoring and the obstructive sleep apnea/hypopnea syndrome, and its prevalence increases with age^20^. Nasal cases are relevant causes of snoring in younger patients, such as the presence of adenoid hypertrophy and nasal septum deviation. Younger patients (<20 years) manifested less snoring compared to older patients at the end of follow-up in our sample (Figure 5). This finding suggests that nasal causes are more relevant etiological factors for snoring in younger patients compared to older patients.

All nasal symptoms regressed in both sexes. Female subjects had more intense postoperative sneezing bouts (Figure 6). Facial pain was also more intense preoperatively, and on the 14^th^ and 60^th^ postoperative days in females (Figure 7). The severity of nasal septum deviation does not correlate with any improvement in nasal obstruction or qualify of life^1^. We found no relationship between nasal septum deviation grade and postoperative improvement in nasal obstruction.

## CONCLUSION

All nasal symptoms in patients undergoing septoplasty with or without partial inferior turbinectomy improve, especially between the preoperative period and the 7^th^ postoperative day. Nasal obstruction improved in about 94% of patients between the preoperative period and the 60^th^ postoperative day.

Nasal obstruction improved markedly in patients with and without allergy symptoms. This improvement was more abrupt in allergic patients on the first follow-up days, while patients without allergies improved later one; both become similar on the 60^th^ postoperative day.
